# Subhepatic Appendicitis With Appendicular Diverticulitis: A Rare Combination for Acute Abdomen

**DOI:** 10.7759/cureus.68754

**Published:** 2024-09-05

**Authors:** Midhun Mathew, Sandra Hembrecht, Kasi Visalatchi Subramanian, Colm Power

**Affiliations:** 1 Department of Surgery, Beaumont Hospital, Dublin, IRL; 2 Department of Surgery, RCSI (Royal College of Surgeons in Ireland) University of Medicine and Health Sciences, Dublin, IRL

**Keywords:** appendicular diverticulitis, laparoscopy, midgut malrotation, subhepatic appendicitis, surgical case report

## Abstract

Subhepatic appendicitis is an unusual presentation of acute appendicitis (AA). Similarly, another uncommon condition that resembles AA is appendiceal diverticulitis (AD), which is a rare form of vermiform appendix pathology. It is exceedingly uncommon for the two to occur simultaneously. We present the case of a 41-year-old male presented with a one-day history of sudden onset of right iliac fossa (RIF) pain associated with a two-day history of nausea and fevers. The only notable lab finding was elevated C-reactive protein (CRP). Clinical examination revealed right abdominal and renal angle tenderness, with RIF rebound and guarding. Computed tomography (CT) concluded acute uncomplicated appendicitis with a subhepatic appendix and he was planned for an emergent laparoscopic appendicectomy. Exposure of the retrocaecal appendix with the caecum in the right loin posed a challenging laparoscopy. The appendix was found to be adherent to the duodenum, right kidney, and transverse colon, and the decision was made to convert to laparotomy to establish safe mobilisation from the duodenum. The appendix was resected in two parts and the histopathology revealed an appendiceal diverticulum with subserosal abscess formation. The subhepatic position of the cecum and appendix is a result of foetal gut malrotation. There is no standard approach for the best course of treatment. The laparotomy conversion gave us better tactile input and direct access to the appendix. Our goal is to educate readers on how to manage an unusual presentation of AA.

## Introduction

There is a wide range of differential diagnoses for acute abdomen, including extremely rare causes, uncommon combinations of different disorders, and anatomic variations. Acute appendicitis (AA), which affects about 7% of the population, is the most common cause of surgical acute abdomen globally [1]. One such uncommon cause of AA is subhepatic appendicitis (SA), which accounted for just 0.08 % of 7210 patients in a 2007 study by Palanivelu et al. [2]. Similarly, a rare but serious form of appendiceal disease that can resemble AA is appendicular diverticulitis (AD). AD is involved in 0.004-2.1% of vermiform appendix pathologies [3].

AA is typically associated with right iliac fossa (RIF) pain, nausea, vomiting, fever and anorexia; however, only 50% of individuals present with these classical symptoms [4]. The clinical picture may be distorted and the initial diagnosis of appendicitis may become more difficult in the event of an anatomical variant where the appendix is positioned abnormally. Whilst nausea, vomiting, and fever are common symptoms of both conditions, there are noticeable differences in their clinical manifestations. SA is linked to pain and tenderness in the right upper quadrant (RUQ), whereas AD is associated with pain and tenderness in the RIF.

Computed tomography (CT) is a commonly used imaging modality to diagnose AA in a setting where physical examination is not deemed conclusive. It is increasingly becoming the modality of choice due to its high levels of sensitivity (88-100%) and specificity (92-98%) [5]. Following diagnosis, surgery is usually the recommended course of action to prevent further complications. Surgery is the gold standard of definitive management for AA as demonstrated by the Conservative Versus Operative Management of Acute Uncomplicated Appendicitis (COMMA) trial [6].

A combination of SA with AD is extremely rare and this report could potentially be the first to present a combined occurrence of both conditions.

## Case presentation

A 41-year-old male presented with a one-day history of sudden onset RIF pain associated with a two-day history of nausea and fever. There was no other significant medical or surgical history. Laboratory results were notable for elevated C-reactive protein and all other blood tests were unremarkable (Table 1).

**Table 1:** Lab results with reference range/normal values.

Lab Parameters	Obtained values	Reference ranges	Unit
Haemoglobin	15.2	13.8-17.2	g/dL
White cell count	8.51	3.5-10.5	x 10^9^/L
Neutrophils	6.59	2.0 - 7.5	× 10^9^ /L
C-reactive protein	55.4	0-5	mg/L
Urea	4.5	3.0–8.0	mmol/L
Creatinine	85	62–106	μmol/L

**Figure 1:**
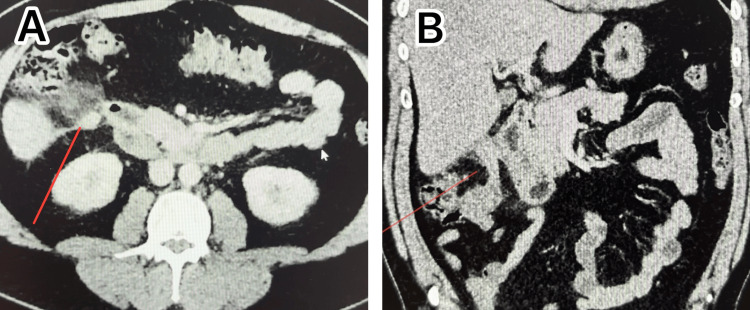
CT findings showing (A) the axial view with the red line pointing towards the appendiceal inflammation and (B) coronal view with red line pointing towards subhepatic appendix

Clinical examination revealed RIF abdominal and renal angle tenderness. Initially, there was RIF rebound tenderness and progressed to localised guarding. The patient was started on intravenous piperacillin/tazobactam and metronidazole. CT of the abdomen and pelvis with IV contrast demonstrated acute uncomplicated appendicitis with an appendix arising medially from the caecum and extending superiorly, with the tip just medial to the inferior right liver lobe (Figure 1).

It was dilated up to 20 mm near the base and tip, with peri appendiceal fat stranding and slightly prominent ileocolic lymph nodes. A laparoscopic appendectomy was performed after obtaining informed consent. All relevant risks were discussed before the patient was transferred to the operating room with the possibility of the need to proceed to open surgery.

**Figure 2:**
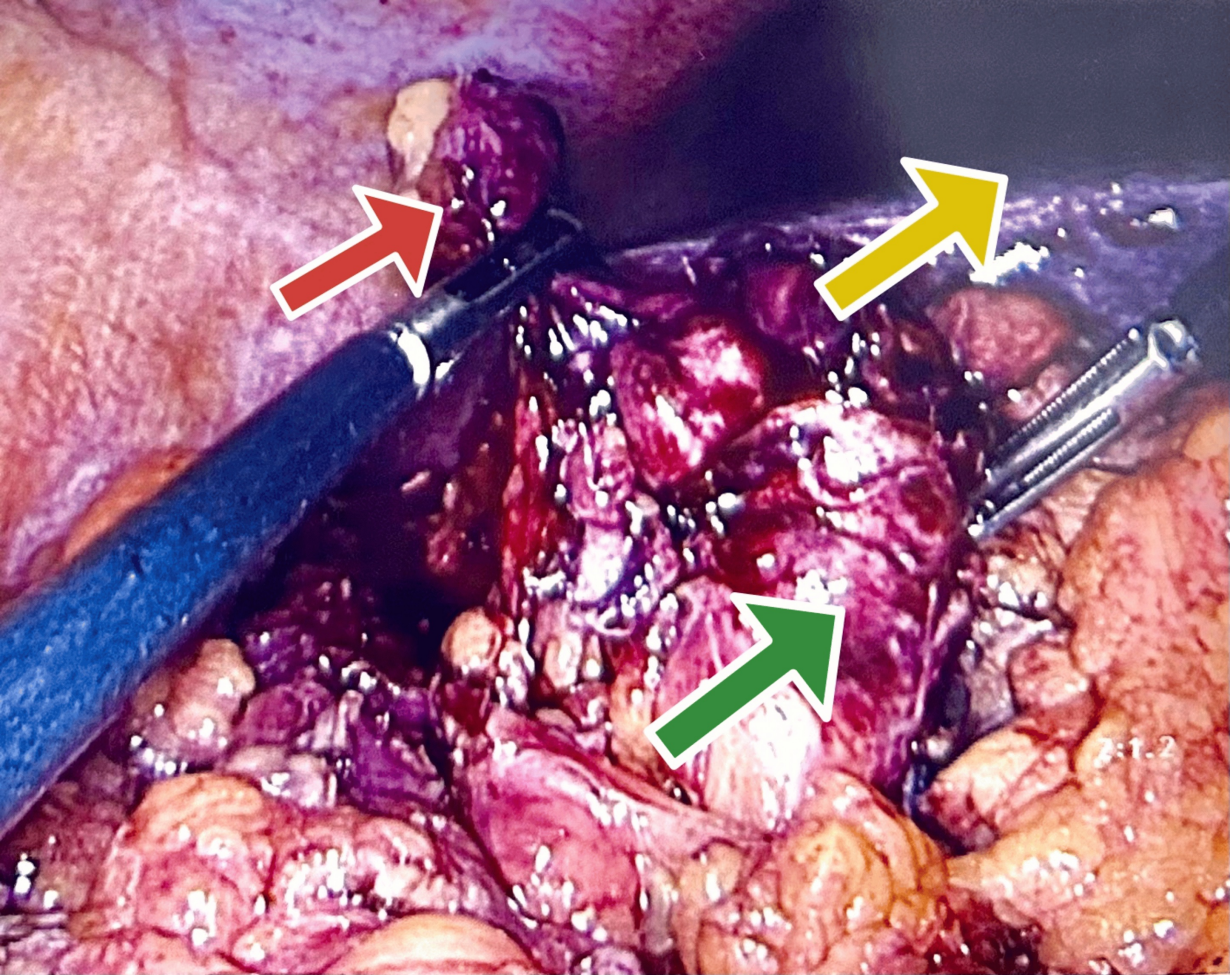
Laparoscopic findings showing subhepatic appendix (red arrow), transverse colon (green arrow), anf the liver surface (yellow arrow)

Exposure of the retrocaecal appendix with the caecum in the right paracolic gutter was challenging laparoscopy. After the infra umbilical port (10 mm), suprapubic (5 mm), left iliac fossa (5 mm), and right upper quadrant (5 mm) ports were inserted to facilitate mobilisation of the appendix safely. Laparoscopic mobilisation of the hepatic flexure, right colon, and caecum was performed. The appendix was found to be adherent to the duodenum, right kidney, and transverse colon and the decision was made to convert to an open procedure via an upper midline laparotomy to establish safe mobilisation from the duodenum (Figure 2).

**Figure 3:**
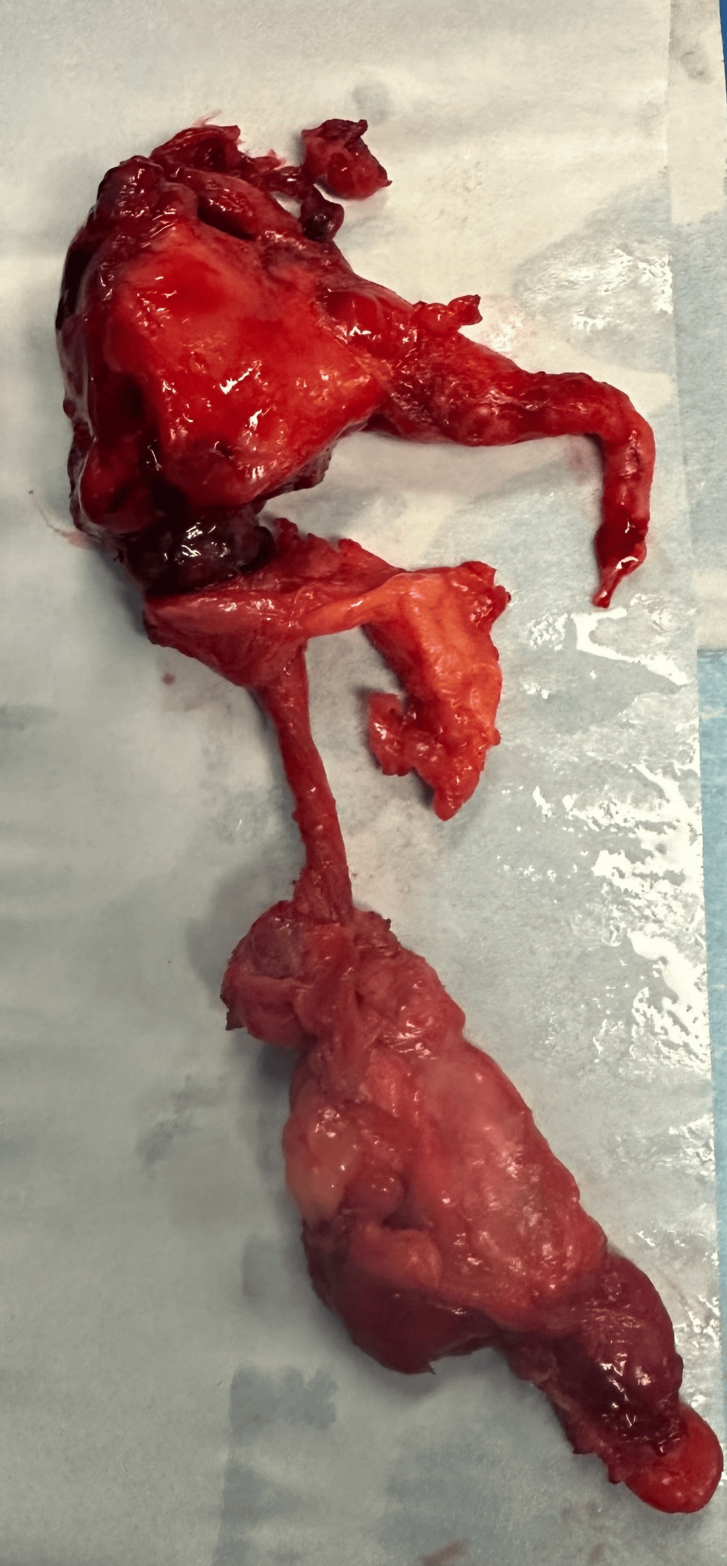
Appendix specimen ressected in two parts

The appendix was dissected out from the underlying duodenum and resected in two parts which were sent for histopathology (Figure 3).

There were two Robinson drains left in the right upper quadrant and suprapubic areas. There were minimal fluid collections (<10 ml) and the drains were removed on postoperative day 3. 

Histopathology revealed a 15 mm appendix with an appendiceal diverticulum and subserosal abscess formation. The patient had an uncomplicated postoperative recovery and was discharged on day 9 with oral antibiotics. 

## Discussion

The most frequent abdominal surgical emergency worldwide is AA, which affects 96.5-100 adults per 100,000 each year [7]. Subhepatic caecum and appendix were first mentioned in 1863, according to a 1955 review by King [8]. Embryologically, the vermiform appendix and cecum form from the caecal bud of the midgut loop. Midgut with its mesentery herniates into the umbilical cord during the sixth week of intrauterine life where it rotates 90 degrees around the axis formed by the superior mesenteric artery (SMA). During the 10th week, the herniated midgut retracts into the abdomen and the caecum with appendix will be located in the sub-hepatic position. A final 180-degree rotation around the axis of the SMA displaces the caecum and appendix into the RIF. Incomplete intestinal rotation or malrotation around the axis of SMA prevents the descent of the caecum and appendix leading to its subhepatic position [9].

Patients with SA usually present with fever, nausea, pain and tenderness in the RUQ mimicking pathology of gastric or hepatobiliary origin [10]. Although our patient presented with both nausea and fever, pain and tenderness were in the RIF and right loin. This could represent a right-sided inflammatory process associated with an ascending retrocaecal appendix. 

Unlike traditional appendicitis, AD is a unique disease process with a different presentation. Localised or generalised peritonitis can be the initial symptom of appendicitis, whereas AD is characterised by a chronic inflammatory process contained within the mesoappendix. Interestingly, this was the first and only episode of similar symptoms in the current case. According to Deschenes et al. [11], males over 30 are typically affected with AD and this is consistent with our patient. Histopathological analysis following appendectomy typically indicates the diagnosis of AD, and our case report was no exception. Our case meets the criteria for microscopic type 4, diverticulum with appendicitis, according to Lipton et al.'s classification of appendiceal diverticular diseases [12].

It is imperative to emphasise the critical role that radiological imaging plays in the diagnosis of both conditions. CT is a highly sensitive modality that can easily detect thickened appendix, inflamed peri appendiceal fat, collections and the presence of free gas in a ruptured appendix. An acutely inflamed ascending retrocecal appendix can cause inflammatory changes that spread to the subhepatic areas. This was observed in our case with a retrocaecal appendix arising medially from the caecum and extending to the inferior right liver lobe with peri appendiceal fat stranding. A retrospective review by Ito et al. demonstrated an absence of fluid level (in the lumen) and appendicolith and the presence of localized abscess formation for patients with AD [13]. Although this wasn’t observed with our patient, it is essential to be aware of this finding for a differential diagnosis of AA.

Considering the uncommon nature of the condition, there is no standard approach or globally recognised consensus for the best course of treatment once the diagnosis has been made. Early surgical intervention is necessary to prevent higher morbidity and mortality due to perforation and peritonitis in AA. SA is associated with a delay in diagnosis due to its location causing consequential problems like sepsis, suppuration, and perforation [10]. AD is associated with a high risk of perforation (which is more than four times higher than that of AA), risk of carcinoid and mucinous malignancies, peritoneal seeding, and pseudomyxoma peritonei [12]. AA is estimated to have a 6.6% perforation rate; however, when a diverticulum is present, that rate increases to 27%. AD leads to an increase in the incidence of appendiceal neoplasms to 26.94% from 1.28% [14]. An accurate diagnosis of both conditions with prompt surgical intervention is essential.

Given the complexity of the case, an exploratory laparoscopy with appendectomy was carried out. Although the procedure was started laparoscopically, the dense duodenal adhesions made it an unsafe option. The laparotomy conversion provided better tactile input and direct access to the appendix whilst ensuring the safety of surrounding structures and allowing thorough assessment for any iatrogenic injury to the underlying duodenum, which had reassuringly not occurred laparoscopically.

A literature search was carried out in the MEDLINE (Medical Literature Analysis and Retrieval System Online) database and yielded no results for a combination of both SA and AD, making our case report quite possibly the first to document a combined occurrence of both conditions. 

## Conclusions

SA and AD are two uncommon pathologies that need prompt diagnosis and optimal treatment. When treating patients who have pain or tenderness in the RUQ or right lower quadrant, SA and AD should be taken into account as differentials, respectively. This case draws attention to a rare instance of SA in which the appendix was anatomically located in the RUQ but the pain was localised to the RLQ. Superior imaging modalities such as an abdominal CT scan combined with a high degree of clinical suspicion can help in the diagnosis and appropriate intervention. To acquire a suitable pathological examination, a precise appendectomy must be carried out as well. The pathology specimen in our case showed AD, making our case report the first with a combination of SA anatomically and AD in the final pathological diagnosis.

The uncommon nature of both conditions makes it difficult for clinicians to diagnose and treat patients in the best possible way. Our goal in sharing this case report is to educate readers on how to manage an unusual presentation of AA.
